# AvrA Exerts Inhibition of NF-κB Pathway in Its Naïve *Salmonella* Serotype through Suppression of p-JNK and Beclin-1 Molecules

**DOI:** 10.3390/ijms21176063

**Published:** 2020-08-23

**Authors:** Chao Yin, Zijian Liu, Honghong Xian, Yang Jiao, Yu Yuan, Yang Li, Qiuchun Li, Xinan Jiao

**Affiliations:** 1 Key Laboratory of Prevention and Control of Biological Hazard Factors (Animal Origin) for Agri-Food Safety and Quality, Ministry of Agriculture of China, Yangzhou University, Yangzhou 225009, China; DX120180114@yzu.edu.cn (C.Y.); MZ120181147@yzu.edu.cn (H.X.); 2 Jiangsu Key Lab of Zoonosis/Jiangsu Co-Innovation Center for Prevention and Control of Important Animal Infectious Diseases and Zoonoses, Yangzhou University, Yangzhou 225009, China; MZ120170979@yzu.edu.cn (Z.L.); d14051@yzu.edu.cn (Y.J.); MX120170779@yzu.edu.cn (Y.Y.); 3 Joint International Research Laboratory of Agriculture and Agri-Product Safety, Yangzhou University, Yangzhou 225009, China; MX120170799@yzu.edu.cn

**Keywords:** AvrA, *Salmonella*, anti-inflammatory response, p-JNK, Beclin-1, proinflammatory cytokines, IL-8

## Abstract

Avian salmonellosis caused by *Salmonella enterica* serovar Enteritidis (*S*. Enteritidis) and Pullorum (*S.* Pullorum) remains a big threat to the poultry industry and public hygiene. AvrA is an effector involved in inhibiting inflammation. Compared to AvrA from *S*. Enteritidis (SE-AvrA), the AvrA from *S.* Pullorum (SP-AvrA) lacks ten amino acids at the C-terminal. In this study, we compared the anti-inflammatory response induced by SP-AvrA to that of SE-AvrA. Transient expression of SP-AvrA in epithelial cells resulted in significantly weaker inhibition of NF-κB pathway activation when treated with TNF-α compared to the inhibition by SE-AvrA. SP-AvrA expression in the *S*. Enteritidis resulted in weaker suppression of NF-κB pathway in infected HeLa cells compared to SE-AvrA expression in the cells, while SP-AvrA expressed in *S.* Pullorum C79-13 suppressed NF-κB activation in infected HeLa and Caco 2 BBE cells to a greater extent than did SE-AvrA because of the higher expression of SP-AvrA than SE-AvrA in *S.* Pullorum. Further analysis demonstrated that the inhibition of NF-κB pathway in *Salmonella*-infected cells corresponded to the downregulation of the p-JNK and Beclin-1 protein molecules. Our study reveals that AvrA modifies the anti-inflammatory response in a manner dependent on the *Salmonella* serotype through inhibition of NF-κB pathway.

## 1. Introduction

Salmonellosis is considered one of the most reported food-borne disease worldwide [1]. *Salmonella enterica* is the etiological agent that causes infection in humans and animals. *Salmonella* uses the type-three secretion system (T3SS) to inject bacterial effector proteins into host intestine epithelial cells to stimulate inflammation, which is crucial for bacterial growth within the intestine [2]. However, it is generally accepted that *Salmonella* can use many effectors (AvrA, SspH1, SptP, and GogB) to prevent or reduce the inflammatory response produced by the host to block the recruitment of phagocytic leukocytes and protect the bacteria from host immune system attack. A strong inflammatory response can kill epithelial cells and macrophages and is permissive for bacterial survival in the host, while chronic intracellular infection maintains a stable niche for bacteria [3]. Activation of the NF-κB pathway promotes the increased expression of many cytokines and chemokines involved in inflammation and immune response. Wild-type *Salmonella* strains activate the NF-κB pathway, whereas nonvirulent or some *Salmonella* serotype strains (*S.* Typhimurium PhoP^c^, *S.* Pullorum) prevent the activation of the NF-κB pathway to attenuate the innate immune responses of the host [4,5].

*Salmonella* uses the T3SS to secret effectors that reverse the activation of signaling pathways involved in the inflammatory response. AvrA is one of the effectors that plays a critical role in inhibiting inflammation and epithelial apoptosis to enhance bacterial proliferation in host cells [4,5,6,7,8,9,10]. AvrA exerts an anti-inflammatory response through the prevention of NF-κB and JNK pathways [6]. The deletion of *avrA* in *Salmonella* induces increased intestinal inflammation, more intense systemic cytokine responses, and increased apoptosis in epithelial cells [3]. AvrA functions as an immune mediator preventing the host from generating an aggressive inflammatory response, and AvrA inhibits apoptosis of infected host cells, allowing for a longer intracellular survival time for *Salmonella* [11]. In addition, AvrA is a multi-functional protein having deubiquitinase and acetyltransferase activity. The AvrA protein deubiquitinates IκBα and β-catenin, blocking the degradation of the two molecules and leading to the inhibition of the NF-κB pathway. Target genes of the NF-κB pathway, such as IL-8 and IL-6, are correspondingly downregulated in the *Salmonella*-infected epithelial cells [5]. Acetylation of MAPKK by AvrA blocks MAPKK phosphorylation activities, leading to inhibition of the downstream JNK and NF-κB pathways [12].

Until now, most studies on the function of AvrA have focused on *S.* Typhimurium; few studies were performed on *S.* Enteritidis [13] and poultry-restricted *Salmonella* serotype Pullorum. Interestingly, protein sequence analysis shows that AvrA in *S.* Pullorum strains (SP-AvrA) lost ten amino acids at the C-terminal of the protein because of the loss of a single nucleotide in the DNA sequence (Figure S1). To reveal the influence of the truncated SP-AvrA in *S.* Pullorum, the anti-inflammatory response induced by SP-AvrA in vitro and in vivo were compared with AvrA from *S.* Enteritidis (SE-AvrA). In addition, the inhibition ability of JNK and Beclin-1 pathways involved in NF-κB activation and anti-inflammatory responses were compared between SP-AvrA and SE-AvrA in epithelial cells infected with *S.* Pullorum and *S.* Enteritidis, respectively.

## 2. Results

### 2.1. SP-AvrA Loss Ten Amino Acids at the C-Terminal of SE-AvrA

Genomic analysis has revealed that many genes are pseudogenized or truncated in *S.* Pullorum compared to *S.* Enteritidis [14]. We compared the AvrA sequences in *S.* Pullorum with that in other closely related serotypes, including *S.* Typhimrium, *S.* Enteritidis, and *S.* Gallinarum (Figure S1). The results demonstrate only AvrA in *S.* Pullorum loses ten amino acids at the C-terminal end of the protein in other serotypes. Additionally, we analyzed AvrA sequences in 96 sequenced *S.* Pullorum isolates, and the results confirm that the AvrA in all of the detected *S.* Pullorum strains lose the C-terminal ten amino acids (Figure S1). To identify the effect of SP-AvrA on anti-inflammatory response, the reference strain C79-13 representing the dominant lineage of *S.* Pullorum based on SNP analysis of core-genome sequences was used in the construction of *avrA* mutants and complementary strains [14]. *S.* Enteritidis P125109 was used as the control strain with complete AvrA sequence. The SP-AvrA and SE-AvrA was transformed into mutants to produce respective complementary strains (Table 1). 

### 2.2. SP-AvrA Displays Weaker Inhibition of NF-κB Activation in Transfected Cells Induced with TNF-α Compared to SE-AvrA

**Figure 1:**
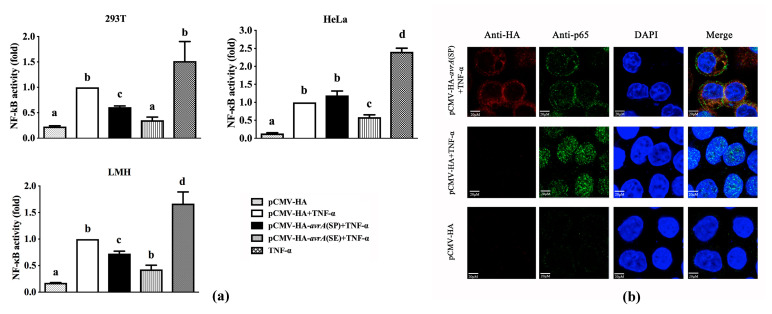
Transient expression of SP-AvrA inhibits the TNF-α activation of NF-κB in transfected cells. (**a**) Dual NF-κB reporter gene assays were performed in HeLa, 293T, and LMH cells cotransfected with plasmids pGL4.32, pRL-TK, and pCMV-HA or expression plasmid for SP-AvrA or SE-AvrA and stimulated with TNF-α (15 ng/mL) for 5 h. Data are expressed as the mean + SD and are shown as fold changes of renilla luciferase activity in cells transfected with pCMV-HA and induced by TNF-α (set as 1). The different letters on the on the error bars indicate statistically significant differences (*p* < 0.05). (**b**) Confocal microscopy of p65 (green), HA-SP-AvrA (red), and nucleus of HeLa cell (blue) was performed in HeLa cells transfected with pCMV-HA-*avrA*(SP) and stimulated with TNF-α (15 ng/mL) for 30 min. The cells transfected with pCMV-HA were used as a positive control for p65 nuclear translocation. Expression of SP-AvrA in transfected HeLa cells inhibits translocation of p65 from the cytoplasm into the nucleus.

The dual NF-κB reporter gene assays were performed to compare the inhibition of NF-κB activation in cells transfected with eukaryotic expression vectors carrying the *avrA* gene at the induction of TNF-α between SP-AvrA and SE-AvrA. First, two plasmids, pCMV-HA-*avrA*(SP) and pCMV-HA-*avrA*(SE), were constructed and identified to be expressed in 293T cells transfected with these plasmids using indirect immunofluorescence (Figure S2). Afterward, three cell lines were used in the dual NF-κB reporter gene assays: HeLa, 293T, and avian hepatocellular carcinoma epithelial cell line LMH (ATCC). Cells transfected with NF-κB reporter gene and *avrA* expressing plasmids were subjected to the detection of renilla luciferase activation. The results show that both SP-AvrA and SE-AvrA inhibit the activation of NF-κB induced by TNF-α, but SE-AvrA is significantly stronger than SP-AvrA at inhibiting NF-κB activation in all three cell lines (Figure 1a). To further identify whether the NF-κB pathway is inhibited by SP-AvrA, the NF-κB subunit p65 nuclear translocation was monitored using confocal microscopy. As shown in Figure 1b, transfected of HeLa cells with pCMV-HA-*avrA*(SP) significantly inhibits the translocation of p65 into nucleus; the nucleuses were occupied by p65 in the TNF-α treated cells.

### 2.3. SP-AvrA Exhibits Weaker Inhibition of Proinflammatory Cytokine Secretion in HeLa Cells Infected by S. Enteritidis Compared to SE-AvrA

**Figure 2:**
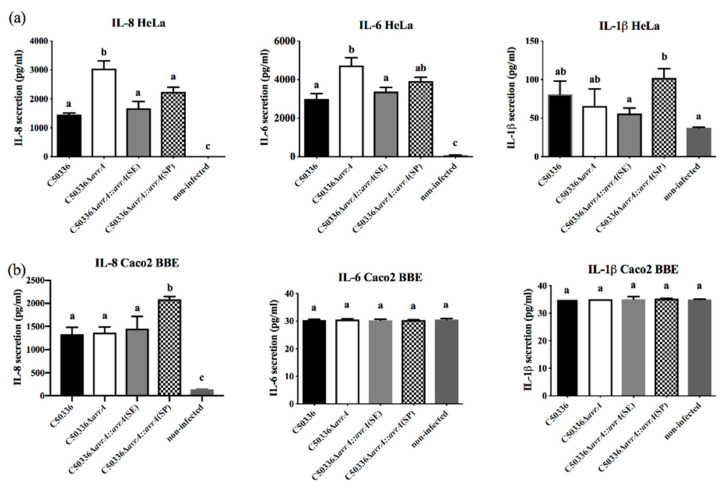
SP-AvrA exhibits weaker inhibition of proinflammatory cytokine secretion in HeLa cells infected by *S*. Enteritidis compared to SE-AvrA. The secreted protein levels of proinflammatory cytokines (IL-8, IL-6, and IL-1β) were measured in the supernatant of HeLa (**a**) or Caco2 BBE (**b**) cells infected with different *S.* Enteritidis strains after 7 h by using FACs according to the instruction of the Human Inflammatory Cytokines Kit (BD, Franklin Lakes Campus, NJ, USA). Data are expressed as the mean + SD. The different letters on the error bars indicate statistically significant differences (*p* < 0.05).

First, the detection time-point of secreted cytokines was determined in the infected HeLa cells. At 4 h post-infection (p.i.), no significant difference was detected in the six cytokines secreted by infected HeLa cells between the C50336 and C50336Δ*avrA* groups (Figure S3). However, at 8 h p.i., the secreted IL-8 and IL-6 were significantly higher in C50336Δ*avrA*-infected HeLa cells than in the wild-type (WT) group (Figure S3). Thus, 8 h p.i. was determined to be the best time-point for cytokines detection. As shown in Figure 2a, the deletion of *avrA* in C50336 caused higher secretion of IL-8 and IL-6 in infected HeLa cells than that in the C50336-infected cells, but the recovery of SP-AvrA or SE-AvrA caused a decreased secretion of IL-8 and IL-6 in *S*. Enteritidis-infected HeLa cells and cells infected with the WT strain, but the SE-AvrA exhibited higher inhibition ability of IL-1β than SP-AvrA. However, deletion of *avrA* in *S.* Enteritidis did not affect the cytokine secretion in infected Caco2 BBE cells (Figure 2b), and *S.* Enteritidis did not induce strong proinflammatory cytokine secretion in Caco2 BBE cells.

### 2.4. SP-AvrA Suppresses IL-8 Secretion in S. Pullorum C79-13-Infected Cells to a Greater Extent than Does SE-AvrA

**Figure 3:**
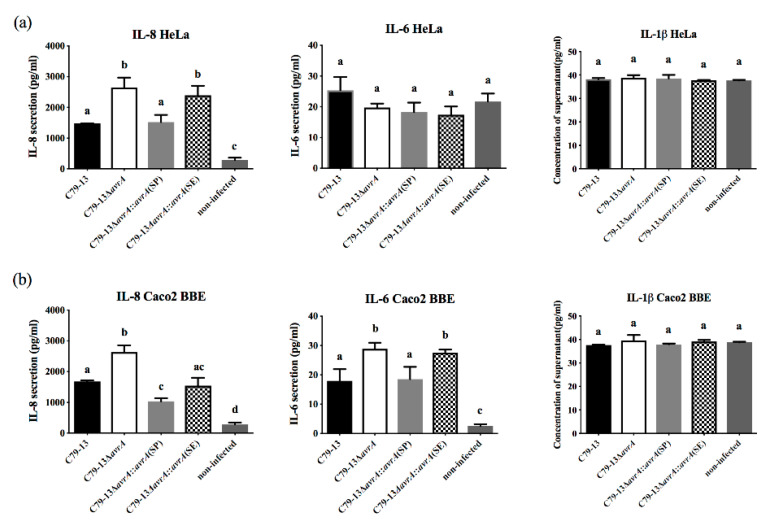
SP-AvrA suppresses IL-8 secretion in *S.* Pullorum C79-13-infected cells to a greater extent than does SE-AvrA. Secretion of IL-8, IL-6, and IL-1β was measured in HeLa (**a**) and Caco2 BBE cells (**b**) infected with *S.* Pullorum C79-13, ∆*avrA* mutant, and two complementary strains using FACs according to the instruction of the Human Inflammatory Cytokines Kit (BD, Franklin Lakes Campus, NJ, USA). Data are expressed as the mean + SD. The different letters on the error bars indicate statistically significant differences (*p* < 0.05).

In C79-13 infected HeLa cells (Figure 3), IL-8 was secreted at higher concentrations in the Δ*avrA* group than in the WT and two complementary strains, but SP-AvrA showed stronger inhibition than SE-AvrA. No difference in IL-6 secretion was detected among the four strains of infected groups. In C79-13 infected Caco2 BBE cells (Figure 3), the secretion of IL-8 was the highest in the Δ*avrA* group among all of four groups, and SP-AvrA remained stronger at suppressing the IL-8 secretion compared to SE-AvrA. The IL-6 secretion in infected Caco2 BBE cells was as low as in HeLa cells, and no significant differences were detected among the four infected groups (Figure 3).

### 2.5. The Effect of SP-AvrA Is Closely Related to Its Role in the JNK and Beclin-1 Pathways

**Figure 4:**
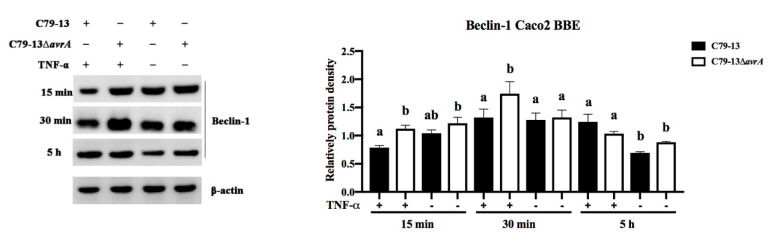
SP-AvrA inhibited Beclin-1 expression in *S.* Pullorum-infected Caco2 BBE cells induced by TNF-α. Caco2 BBE cells were infected with *S.* Pullorum C79-13 and C79-13∆*avrA*. After 1 h incubation, the cells were treated with TNF-α (15 ng/mL) for 15 min, 30 min, or 5 h and subjected to Western blot analysis of Beclin-1. Protein band density was analyzed by using the NIH ImageJ software. Data are representative of three independent experiments. Data are expressed as the mean + SD. The different letters on the error bars indicate statistically significant differences (*p* < 0.05).

To detect the relationship between the NF-κB pathway and the Beclin-1 molecule, *S.* Pullorum was used to infect Caco2 BBE cells followed by treatment with TNF-α for either 15 min, 30 min, or 5 h. Beclin-1 protein was significantly lower in C79-13-infected Caco2 BBE cells compared to Beclin-1 levels in C79-13Δ*avrA*-infected cells during treatment with TNF-α for 15 min (Figure 4), which implies that SP-AvrA inhibited expression of Beclin-1 in *Salmonella*-infected cells treated with TNF-α.

**Figure 5:**
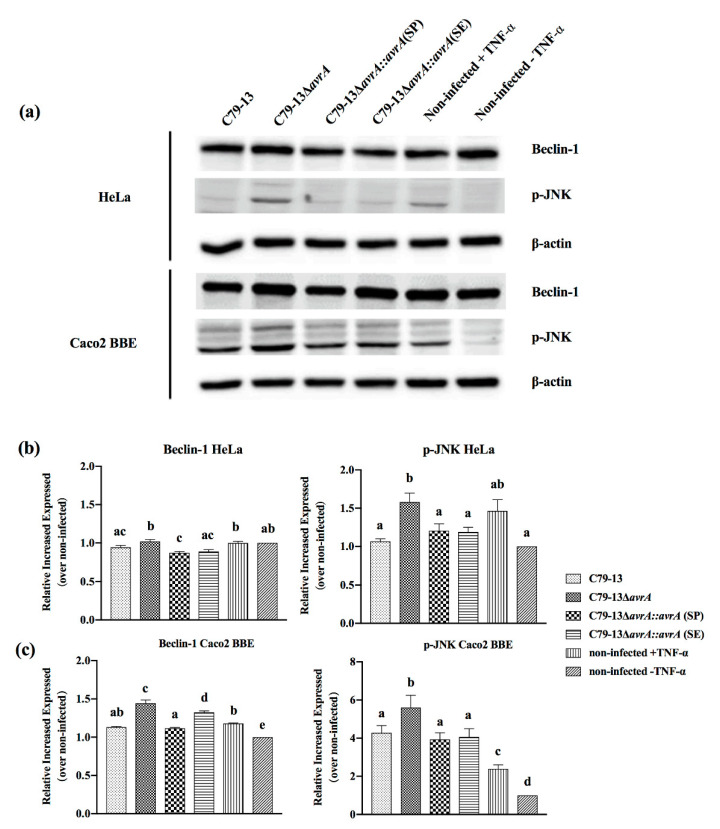
SP-AvrA inhibits the p-JNK and Beclin-1 pathways in *S*. Pullorum-infected cells. HeLa (**b**) or Caco2 BBE cells (**c**) were infected with different *S.* Pullorum strains. After 1 h post-infection and the following 15-min stimulation of TNF-α (15 ng/mL), the cell lysates were subjected to Western blot analysis with anti-p-JNK, anti-Beclin-1, or anti-β-actin antibodies (loading control) (**a**). Protein band density was analyzed by using the NIH ImageJ software. Data are presented as the mean + SD relative to control. The different letters on the error bars indicate statistically significant differences (*p* < 0.05).

To study whether proinflammatory cytokine secretion is related to activation of the NF-κB pathway, the expression levels of p-JNK and Beclin-1 proteins were detected in *S.* Pullorum-infected cells. In C79-13-infected cells, the p-JNK and Beclin-1 protein levels in C79-13Δ*avrA*-infected cells increased significantly in both HeLa and Caco2 BBE cells, and the C79-13Δ*avrA::avrA*(SP) recovered a similar expression level of p-JNK, as well as C79-13, while the p-JNK and Beclin-1 expression level in C79-13Δ*avrA::avrA*(SE) infected cells were higher than in C79-13Δ*avrA::avrA*(SP) groups (Figure 5). In addition, the p-JNK and Beclin-1 expression levels corresponded to the IL-8 secretion of infected cells (Figure 3 and Figure 5).

### 2.6. The SE-AvrA Displays Stronger Inhibition Ability of the JNK and Beclin-1 Pathways in S. Enteritidis-Infected Caco2 BBE Cells than That of SP-AvrA Expressed in S. Enteritidis

**Figure 6:**
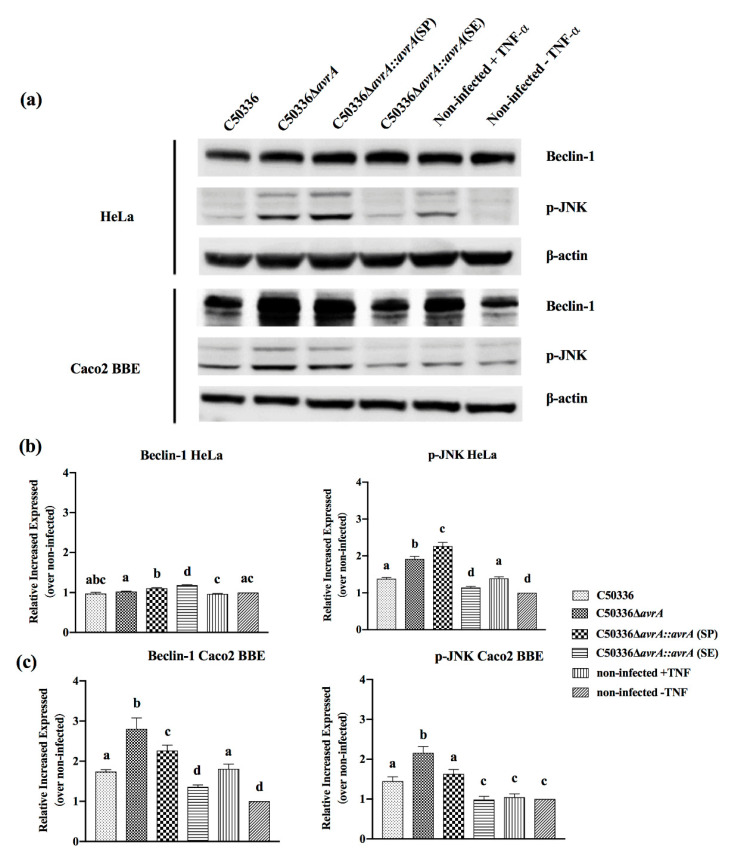
SE-AvrA displays stronger inhibition ability of the p-JNK and Beclin-1 pathways in *S*. Enteritidis-infected cells than SP-AvrA. HeLa (**b**) or Caco2 BBE cells (**c**) were infected with different *S.* Enteritidis strains. After 1 h post-infection and the following 15-min stimulation of TNF-α (15 ng/mL), the cell lysates were subjected to Western blot analysis with anti-p-JNK, anti-Beclin-1, or anti-β-actin antibodies (loading control) (**a**). Protein band density was analyzed by using the NIH ImageJ software. Data are presented as the mean + SD relative to control. The different letters on the error bars indicate statistically significant differences (*p* < 0.05).

In C50336-infected cells, the p-JNK and Beclin-1 protein levels in C50336Δ*avrA*-infected cells increased significantly in Caco2 BBE cells, and the C50336Δ*avrA::avrA*(SE) recovered a similar expression level of p-JNK, as well as C50336, while the p-JNK and Beclin-1 expression level in C50336Δ*avrA::avrA*(SP) infected Caco2 BBE cells were significantly higher than in C50336Δ*avrA::avrA*(SP) group (Figure 6).

### 2.7. The SP-AvrA Dampens Higher Dissemination of S. Pullorum in Mucosal Tissues than That of SE-AvrA

**Figure 7:**
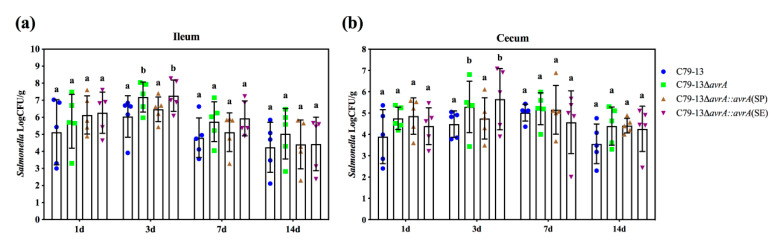
SP-AvrA dampen colonization and dissemination of *S.* Pullorum in mucosal tissues of chicken. Four-day-old HY-Line chickens were infected with *S.* Pullorum strains, and the bacteria from ileum (**a**) and cecum (**b**) were counted on MacConkey agar media plates at 1, 3, 7, and 14 days post infection. Data are expressed as the mean ± SD. The different letters on the error bars indicate statistically significant differences (*p* < 0.05).

Previous studies confirmed that AvrA performed as an anti-inflammatory factor to reduce the self-limited inflammation and decrease the dissemination of *Salmonella* into extracellular tissues, which is beneficial for bacterial persistent infection associated with decreased systemic disease [3,13]. To compare the effect of SP-AvrA and SE-AvrA on the bacterial load of *S*. Pullorum in chicken mucosal tissues, the four-day-old HyLine chickens were inoculated orally with 9 log CFU of the corresponding *S*. Pullorum strain (C79-13; C79-13Δ*avrA*; C79-13Δ*avrA::avrA*(SE)*;* C79-13Δ*avrA::avrA*(SP)). Deletion of *avrA* caused a significantly increased colonization of *S.* Pullorum in both ileum and cecum at three days post infection compared to the WT strain (Figure 7). However, SP-AvrA could induce a decreased bacterial load to a similar level as well as C79-13, which was not detected in C79-13Δ*avrA::avrA*(SE) strain with expressed SE-AvrA (Figure 7).

### 2.8. The Expression of SP-AvrA Is Higher in S. Pullorum than That of SE-AvrA

**Figure 8:**
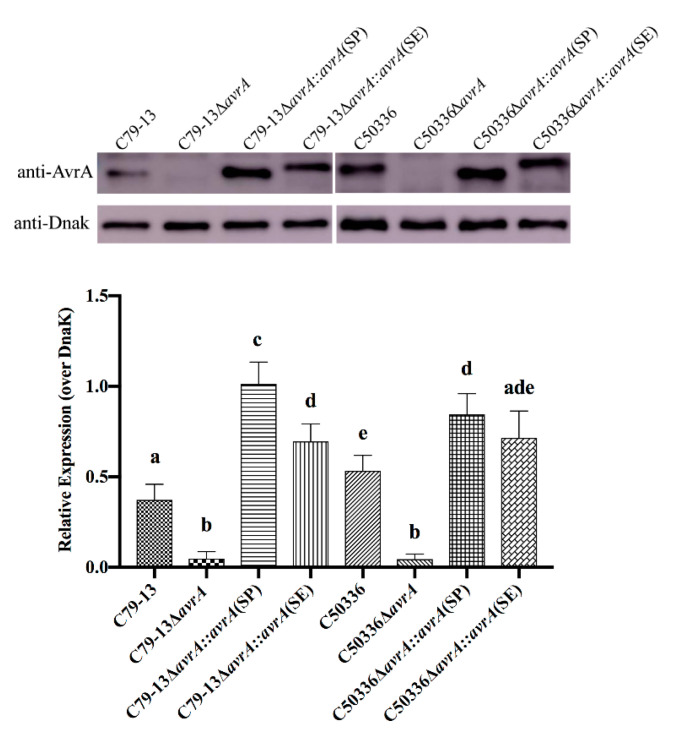
Identification of AvrA expression in C79-13 and C50336 by Western blot analysis. The overnight cultured bacteria were lysed using ultrasonic treatment and subjected to Western blot assay with mouse anti-AvrA sera as the primary antibody. The DnaK was used as the control. Protein band density was analyzed by using the NIH ImageJ software. Data are expressed as the mean + SD. The different letters on the error bars indicate statistically significant differences (*p* < 0.05).

Western blot analysis was used to determine the expression level of AvrA in both C79-13 and C50336. As shown in Figure 8, the AvrA was not detected in both mutant strains, confirming the successful construction of the mutants. Transformation of plasmid carrying *avrA*(SP) into C79-13Δ*avrA* induced significantly higher expression of SP-AvrA than that of SE-AvrA in *S.* Pullorum C79-13Δ*avrA::avrA*(SP). However, the expression level of SP-AvrA showed no significant difference with that of SE-AvrA in *S*. Enteritidis C50336.

## 3. Discussion

During invasive *Salmonella* infection, pattern recognition receptors initiate the innate immune system leading to the recruitment of neutrophils and macrophages and the production of proinflammatory cytokines (IL-6, IL-1β) to promote pathogen clearance [16]. PMNs are important for host resistance to Salmonella colitis. Chemokines such as IL-8 are required for efficient chemotaxis of PMNs across intestinal epithelial cell monolayer during *Salmonella* infection [17]. It is well recognized that wild-type *S.* Typhimurium can induce acute intestinal inflammation, but nonpathogenic *Salmonella* strains such as *S.* Typhimurium PhoP^c^ and *S.* Pullorum attenuate the synthesis of inflammatory effector molecules (IL-8) elicited by a spectrum of proinflammatory stimuli (TNF-α) [18]. Theses strains considered as anti-inflammatory organisms have immunosuppressive effects involving the inhibition of the IκBα/NF-κB pathway by blockade of IκBα degradation through *Salmonella* T3SS effectors, such as AvrA and GogB [4,18,19]. These effects are mediated by blocking IκBα ubiquitination without affecting IκBα phosphorylation [18]. AvrA, a YopJ-like protein, is a multi-function protein involved in the deubiquitinase of IκBα to prevent its degradation, which is essential to activation of NF-κB pathway [4,10]. It also possesses acetyltransferase activity towards MAPKKs and inhibits JNK activation, which is closely related to activation of the NF-κB signaling pathway [6]. The AvrA protein in *S.* Enteritidis enhances the tight junction (TJ) barrier by stabilizing ZO-1 expression in epithelial cells through inhibition of the JNK pathway, thus increasing cell permeability and reducing bacteria invasion and the inflammatory response in mice [13].

Most studies on AvrA function have been performed on *S.* Typhimurium and *S.* Enteritidis; few studies are reported in host-restricted serotype *S.* Pullorum. Interestingly, the AvrA protein in *S.* Pullorum loses ten amino acids at the C-terminal of the protein based on the analysis of 97 sequences of *S.* Pullorum isolates (Figure S1). Transient expression of SP-AvrA in eukaryotic cells (HeLa, 293T, and LMH) inhibits the NF-κB activation induced by TNF-α and AvrA from other *Salmonella* serotypes [4], but the inhibition ability of SP-AvrA is weaker than that of SE-AvrA expressed transiently in these cells (Figure 2), suggesting that the deficiency of the C-terminal ten amino acids may affect the function of AvrA in preventing NF-κB activation. In *S*. Enteritidis, the deletion of *avrA* causes the upregulated expression of the proinflammatory cytokine IL-8 in infected human epithelial cells through the inhibition of the JNK pathway [13]. Complementation of SP-AvrA in a *S*. Enteritidis Δ*avrA* mutant recovers the inhibition of IL-8 secretion in infected HeLa cells, but the ability is reduced compared to SE-AvrA in the mutant strain. It is appropriate for *S*. Enteritidis to use AvrA with the entire sequence to develop the anti-inflammatory response. However, we did not find a significant difference of IL-8 secretion among Caco2 BBE cells infected with *avrA-*positive and negative *S.* Enteritidis strains, and this outcome does not correspond to the previous study [13]. Compared to the function of AvrA in Caco2 BBE cells, the inhibition of IL-8 by AvrA in *S.* Enteritidis-infected HCT116 and SKCO15 is obvious and significant [13]. These differences reflect that AvrA displays its function in the appropriate cells. Additionally, SE-AvrA promotes the expression of IL-8 in both S06004Δ*avrA::avrA*(SE)-infected cells, suggesting that SE-AvrA does not suppress IL-8 secretion in the infected cells. We demonstrated that the difference of immune response caused by AvrA was possibly related to the expression variation of AvrA in different *Salmonella* isolates [20]. The expression of AvrA is controlled by a *Salmonella*-specific regulatory system, which produces phenotypical classes of *Salmonella* strains expressing AvrA in different conditions. Class 1 stains have constitutive synthesis of AvrA, Class 2 strains use an acid for induction of AvrA, and Class 3 strains have silent *avrA* genes [20]. Even in the same serotype of *S.* Typhimurium, the three classes are detected in different strains [20]. Although the AvrA is constitutively expressed in C79-13 and C50336, the expression level for SP-AvrA is higher than SE-AvrA expressed in C79-13, while the expression of SP-AvrA is at the similar level as the SE-AvrA in C50336. However, the SE-AvrA displayed stronger anti-inflammatory effects than SP-AvrA in C50336 (Figure 2).

The suppression of proinflammatory cytokines by AvrA is associated with the inhibition of NF-κB activation mainly through p-JNK pathway [6]; therefore, we compared the p-JNK concentration of infected cells with the results of IL-8 and IL-6 expression levels. The p-JNK concentration was increased in the cells with upregulated IL-8 secretion, and vice versa (Figure 2, Figure 3, Figure 5 and Figure 6). In addition, the Beclin-1 protein level correlated with p-JNK and IL-8 secretion (Figure 2, Figure 3, Figure 5 and Figure 6). Although Beclin-1 is a key regulator for autophagy and apoptosis, activation of autophagy induces an increased expression of the anti-inflammatory cytokine IL-10 and decreases the expression of IL-8 and TNF-α [21]. It is reported that *S.* Enteritidis AvrA inhibits autophagic response by decreasing the Beclin-1 concentration, which may be associated with the JNK/AP-1 signaling pathway [22]. In our study, deficiency of SP-AvrA in *S.* Pullorum C79-13 caused the upregulation of Beclin-1 and p-JNK in infected HeLa and Caco2 BBE cells, suggesting that SP-AvrA may control autophagy in the anti-inflammatory response.

## 4. Materials and Methods

### 4.1. Strains and Plasmids

Strains and plasmids are listed in Table 1. The construction of *avrA* mutants was performed using the λ-RED recombination system [15]. Plasmid pBR322 was used to carry the *avrA* gene and then transformed into *avrA* mutants to produce complementary strains. The pCMV-HA vector was used to construct eukaryotic expression plasmids of AvrA.

### 4.2. Cell Culture and Bacterial Infection Assay

The 293T, Caco2 BBE, and HeLa cells and the chicken hepatocellular carcinoma epithelial LMH cells were routinely maintained in DMEM supplemented with 10% fetal calf serum (ClarkBio, Richmond, VA, USA), penicillin–streptomycin (100 U/mL and 100 µg/mL), and L-glutamine (2 mM). HeLa cells were infected with the indicated *Salmonella* strains at a MOI of 100 for 1 h. The adhesion assay was performed by lysing the cells with 0.1% Triton X100 (Sigma, Saint Louis, MO, USA) and then plating on LB agar. To conduct the invasion assay, the cells were incubated in DMEM supplemented with gentamicin (100 μg/mL) to kill extracellular bacteria; the cells were then harvested for lysis and plated on the LB agar. The infection rate was calculated as percentage of intracellular bacteria to initial bacteria number added to the well.

### 4.3. Dual NF-κB Reporter Gene Assay

Cells were transiently cotransfected with 250 ng of pGL4.32 (Promega, Madison, WI USA), 20 ng pRT-TK (Promega, Madison, WI, USA), and each recombinant plasmid with AvrA in pCMV-HA vectors using Lipofectamine^®^3000 reagent according to the manufacturer’s instructions (Life Technologies, Waltham, MA, USA). After 24 h cultivation, the cells were stimulated by 15 ng/mL TNF-α and incubated in DMEM for 5 h. Luciferase activity was monitored using the dual luciferase assay system (Promega, Madison, WI, USA).

### 4.4. Confocal Microscopy

HeLa cells were transiently transfected with 500 ng pCMV-HA-*avrA*(SE), pCMV-HA-*avrA*(SP), or pCMV-HA plasmids. After 24 h cultivation followed by stimulation with 15 ng/mL TNF-α for 30 min, the cells were fixed for 20 min in 4% paraformaldehyde in PBS, washed in PBS, permeabilized with 0.1% Triton X-100 in PBS for 5 min, and washed again. Fixed samples were incubated in blocking solution (5% BSA in PBS) for 2 h at 37 °C followed by overnight incubation with rabbit anti-p65 (Abcam, Cambridge, UK) and mouse anti-HA (Abcam, Cambridge, UK). The samples were then incubated with goat anti-rabbit IgG (Alexa Fluor^®^ 488; Abcam, Cambridge, UK) and goat anti-mouse IgG (Alexa Fluor^®^ 546, Cambridge, UK) for 5 h and DAPI for 20 min. The coverslips were mounted on glass slides, and stained cells were observed by laser scanning confocal microscopy (Leica Microsystems, Mannheim, Germany).

### 4.5. Detection of Secreted Proinflammatory Cytokines

The proinflammatory cytokines were measured in cell culture medium using the BD Cytometric Bead Array (CBA) Human Inflammatory Cytokines Kit (BD, Franklin Lakes Campus, NJ, USA) according to the manufacturer’s instructions. FACSAria SORP (BD, Franklin Lakes Campus, NJ, USA) was needed for the kit used to determine the concentration of eight cytokines, including IL-8, IL-1β, IL-6, IL-10, TNF-α, and IL-12p70.

### 4.6. Western Blot Analysis

Cell lysates were prepared, electrophoresed on 12% SDS-polyacrylamide gels, and then transferred to PVDF membranes using a Semi-dry transfer device (Bio-Rad, USA). Immunoreactive proteins were detected with antibodies to p-JNK (Abcam, Cambridge, UK) or Beclin-1 (Abcam, Cambridge, UK) using ECL (Thermo Scientific, Waltham, MA, USA) and a goat anti-mouse HRP secondary antibody (Abcam, Cambridge, UK). Blots were exposed to film for 1–2 h in the Amersham Imager 600 device (GE healthcare, Pittsburgh, PA, USA).

### 4.7. Animal Test

To evaluate the effect of AvrA on colonization of *Salmonella* in chickens, 80 4-day-old HyLine White chickens were randomly divided into four groups of 20 chickens, which was immunized orally with 9 log CFU of *S*. Pullorum C79-13, C79-13Δ*avrA*, C79-13Δ*avrA::avrA*(SE)*,* and C79-13Δ*avrA::avrA*(SP), respectively. At 1, 3, 7, and 14 days post immunization, five chickens from each group were killed for collection of liver, spleen, ileum, and cecum samples, which were then subjected to calculate the bacterial number on Brilliant green agar plates.

### 4.8. Statistical Analysis

All of the data were analyzed using GraphPad Prism version 8.0, and one-way ANOVA with Tukey’s multiple comparison test was performed to identify significant differences. A *p* value < 0.05 was considered significantly different between two groups.

## 5. Conclusions

In summary, the loss of ten amino acids on the C-terminal of SP-AvrA transiently expressed in cells stimulated with TNF-α downregulated the inhibition to NF-κB activation compared to that of SE-AvrA. However, both SP-AvrA and SE-AvrA performed their anti-inflammatory responses in their parent bacterial strains. Transformation of SP-AvrA into the *S.* Enteritidis Δ*avrA* mutant did not recover the anti-inflammatory response to the same level as SE-AvrA and vice versa. In addition, SP-AvrA and SE-AvrA exerted varying degrees of inhibition of inflammatory response in *S.* Pullorum because of its different expression level. Further analysis demonstrated that the anti-inflammatory response of AvrA in *Salmonella*-infected epithelial cells was closely associated with the inhibition of the JNK and Beclin-1 pathways.

## Figures and Tables

**Table 1:** Bacterial strains and plasmids.

Stains or Plasmid	Description of Genotype	Source (Reference)
*S.* Enteritidis		
C50336	Wild-type strain	China Institute of Veterinary Drug Control
C50336Δ*avrA*	*avrA* mutant of C50336	[13]
C50336Δ*avrA::avrA*(SE)	Complementary strain with pBR322-*avrA*(SE) in C50336*ΔavrA*	[13]
C50336Δ*avrA::avrA*(SP)	Complementary strain with pBR322-*avrA*(SP) in C50336Δ*avrA*	This work
*S.* Pullorum		This work
C79-13	Wild-type strain	China Institute of Veterinary Drug Control
C79-13Δ*avrA*	*avrA* mutant of C79-13	This work
C79-13Δ*avrA::avrA*(SE)	Complementary strain with pBR322-*avrA*(SE) in C79-13Δ*avrA*	This work
C79-13Δ*avrA::avrA*(SP)	Complementary strain with pBR322-*avrA*(SP) in C79-13Δ*avrA*	This work
*E. coli*		
DH5α	Host cells for pCR2.1, pKD46 and pKD3	Takara
Plasmids		
pKD3	chloramhenicol resistance cassette	[15]
pKD46	Ap^r^, λ-Red mutation system	[15]
pBR322	Ap^r^, vector to construct recombinant complementary plasmid	Invitrogen

## Supplementary Materials

Supplementary Materials can be found at https://www.mdpi.com/1422-0067/21/17/6063/s1.

## Abbreviations

DAPI	4′,6-diamidino-2-phenylindole
LB	Luria–Bertani
PVDF	polyvinylidene fluoride
ECL	Electrogenerated chemiluminescence
