# Supplementary material for: AvrA Exerts Inhibition of NF-κB Pathway in Its Naïve Salmonella Serotype through Suppression of p-JNK and Beclin-1 Molecules
Source: Int J Mol Sci. 2020 Aug 23;21(17):6063. doi: 10.3390/ijms21176063 (PMC7504150; doi:10.3390/ijms21176063)
Supplement: Supplementary file 1 [file ijms-21-06063-s001.pdf]

International Journal of
Molecular Sciences

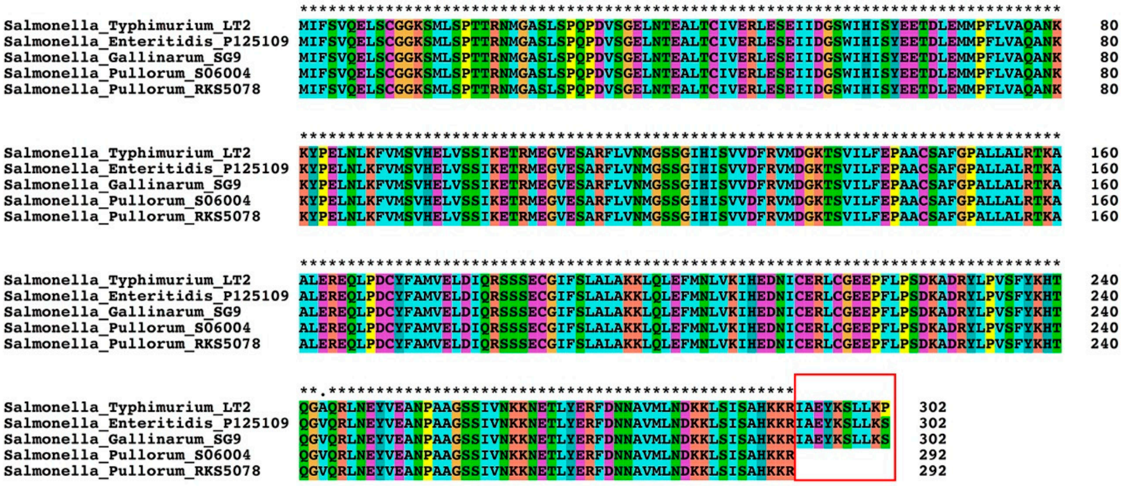

Figure S1. Alignment of AvrA sequences from different *Salmonella* serotypes. The amino acid sequences of AvrA were multi-aligned among *S.* Typhimurium, *S.* Enteritidis, *S.* Gallinarum and *S.* Pullorum strains. The Clustalx 2.1 software was used to perform alignment analysis. The red box displays the ten amino acids lost in the C-terminal of AvrA from *S.* Pullorum.

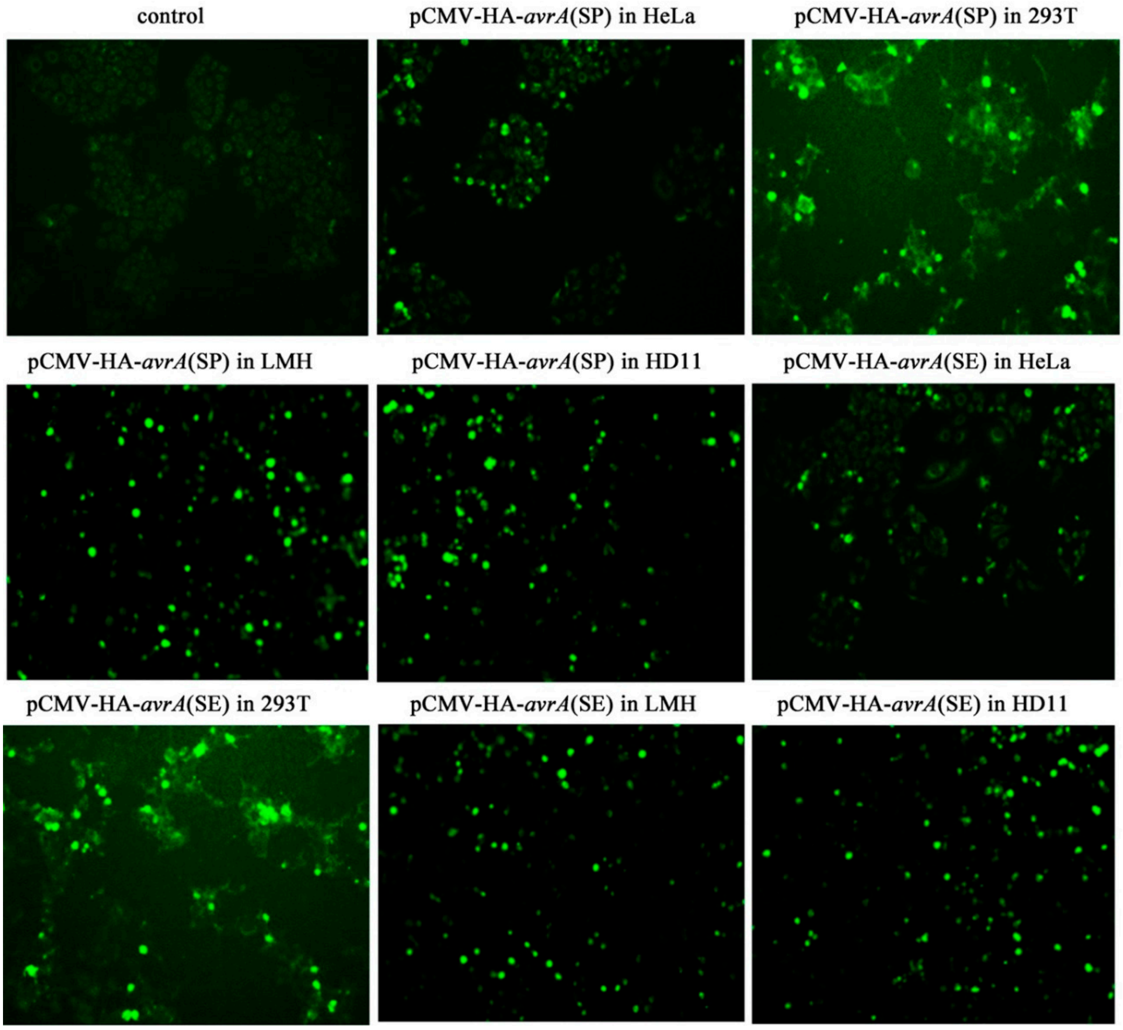

Figure S2. Identification of AvrA expression in cells transfected by pCMV-HA-*avrA*(SP) or pCMV-HA-*avrA*(SE) using indirect immunofluorescence. The eukaryotic expression plasmids pCMV-HA-

avrA(SP) and pCMV-HA-*avrA*(SE) were transfected into different cells (HeLa, 293T, and LMH) and subjected to the indirect immunofluorescence by using Mouse anti-HA antibody and Goat Anti-Mouse IgG H&L (Alexa Fluor® 488). The green fluorescence represents expression of AvrA in transfected cells.

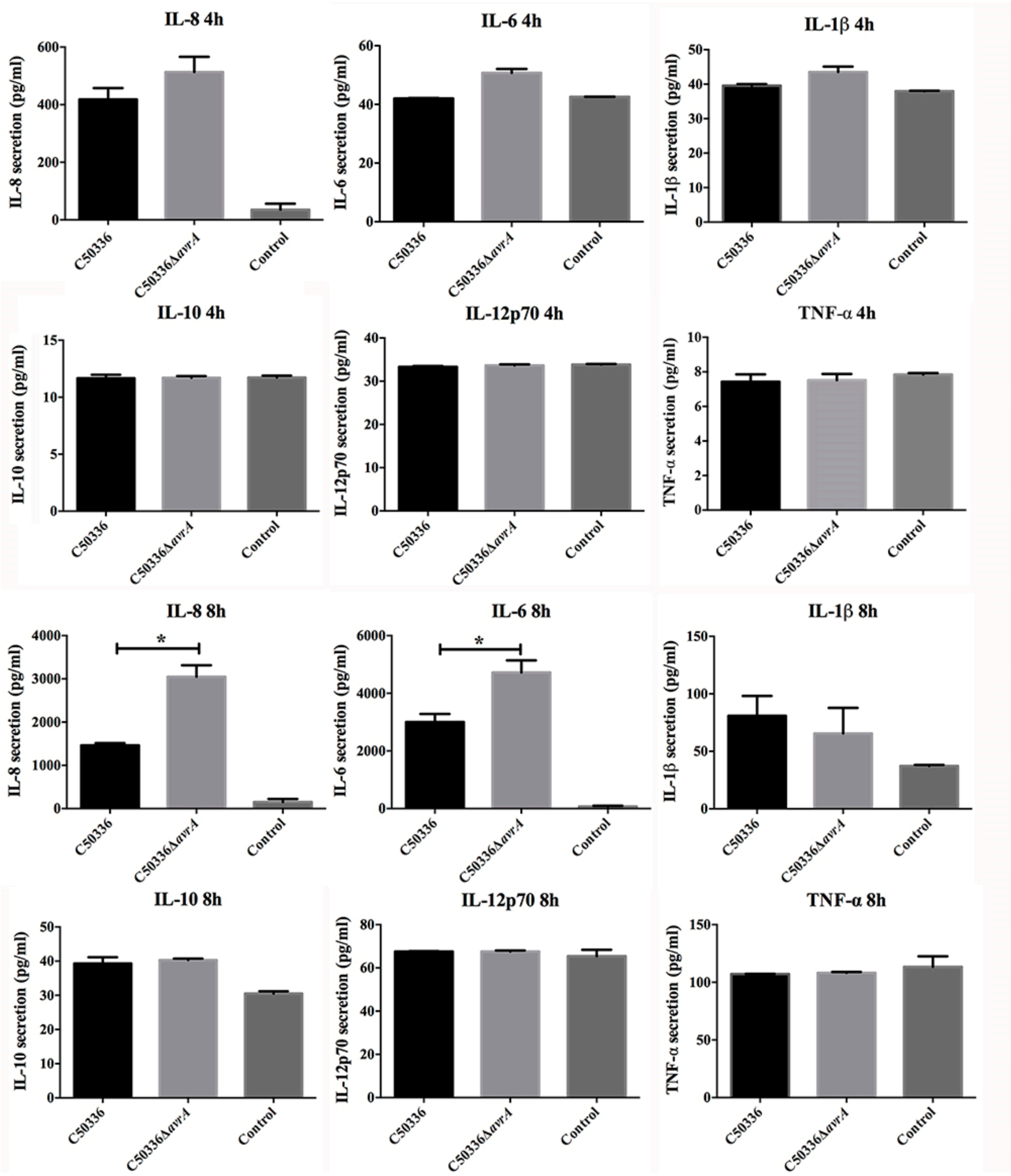

Figure S3. Determination of infection time-point to detect secretion of proinflammatory cytokines in *S.* Enteritidis infected HeLa cells. The secretion of proinflammatory cytokines was measured at 4 h and 8 h after inoculation of HeLa cells with *S.* Enteritidis. At 4 h post-infection (p.i.), there was no difference in secreted cytokines between C50336-infected and C50336Δ*avrA*-infected cells, while IL-8 and IL-6 were significantly higher in C50336Δ*avrA*-infected cells than in cells infected with wild-type strain ("*", $p < 0.05$).
